# Puffer Fish Gut Microbiota Studies Revealed Unique Bacterial Co-Occurrence Patterns and New Insights on Tetrodotoxin Producers

**DOI:** 10.3390/md18050278

**Published:** 2020-05-25

**Authors:** Zhenchi Li, Jinglin Tian, Yukun Lai, Chiu-Hong Lee, Zongwei Cai, Chun-Fai Yu

**Affiliations:** 1 Chemistry Department, Hong Kong Baptist University, Kowloon, Hongkong 999077, China; 17482224@life.hkbu.edu.hk (J.T.); 19448481@life.hkbu.edu.hk (Y.L.); zwcai@hkbu.edu.hk (Z.C.); 2 Environmental Science Department, Beijing Normal University-Hong Kong Baptist University United International College, Zhuhai 519087, China; chiuhonglee@uic.edu.hk

**Keywords:** tetrodotoxin, puffer fish, 16S rRNA, gut microbiota, bacterial symbionts

## Abstract

Tetrodotoxin (TTX) is a potent neurotoxin isolated mainly from toxic puffer fish. To date, the TTX biosynthetic mechanism inside its hosts remains unresolved. Here, we hypothesize the TTX synthesis relies on the host gut microbiota, including the neglected non-culturable bacteria. In these studies, we collected the gut contents from 5 puffer fish species of the genus *Takifugu* including one suspected hybrid species for gut microbiota study by 16S rRNA amplicon metagenomics approach. Their gut samples were divided into toxic and non-toxic groups based on the TTX concentrations in the livers detected by LC-MS/MS. Bacterial diversity studies showed that gut microbiota structures were significantly different between toxic and non-toxic species. *Vibrio* and Cyanobacteria centered at the gut bacterial co-occurrence network, suggesting their importance in TTX biosynthesis. The results of PICRUSt2 metagenomic prediction and gene set enrichment analysis provided new support of arginine-precursor required in TTX biosynthesis. This is the first study to profile the gut microbiota in toxic and non-toxic puffer fish species by 16S rRNA amplicon metagenomic approach, defining significant microbial co-occurrence patterns in their gut environment. Our data supported the proposed biosynthesis of TTX inside the hosts by their gut bacterial symbionts using arginine as a precursor.

## 1. Introduction

Tetrodotoxin (TTX) has been known as a sodium channel blocker for many years, which prevents action potentiation to be generated or propagated in nerve cells [1]. TTX can be used as a local anesthetic [2] and it is a popular research tool in neurophysiological studies [3]. The high demand of TTX in research studies leads to the successful chemical synthesis of TTX and its analogues [4,5,6]. However, the synthetic steps and yield were so complex and low that the current TTX available on the market depends on the direct extraction and purification from toxic puffer fish [7], which are a limited resource and therefore an alternative source is required to meet the demand. 

Biosynthesis of TTX involving enzymes and substrates could prevent the destruction of marine resources [8]. However, the unknown origin and precursors of TTX hampers researchers on elucidating its biosynthetic pathways. TTX containing animals have been identified across different animal phyla and a wild range of trophic levels in the marine environment, such as the Mollusca, Nemertea, Arthropoda, and Chordata [9,10,11,12], suggesting an exogenous source of TTX. Moreover, the discoveries of numerous TTX-producing bacteria further suggested the symbiotic synthesis of TTX inside their hosts [13]. However, the amount of TTX in toxic puffer fish varies amongst individuals within the same species, and inter-species, such as sex, age, and seasons [14,15,16], suggesting both inherent and environmental factors are possible to influence the TTX biosynthesis. On the other hand, some researchers queried the bacterial original of TTX as several studies reported the very low *in-vitro* production of TTX-producing bacteria and even zero detection of TTX in a previously reported TTX-producing bacteria *Vibrio alginolyticus* [17].

The fish gut environment has a reservoir of bacteria that serves as an important functional unit to maintain metabolic homeostasis in the fish [18]. TTX-producing bacteria were commonly isolated from the gut environment of puffer fish [19,20]. Although inherent factors such as signaling trigger or food preference may determine TTX biosynthesis, unique bacterial symbiont in the puffer fish gut is also vital [8]. Therefore, the profiling and comprehensive studies on gut microbiota of TTX containing species such as puffer fish are significant in the discovery of key factors or biomarkers in TTX biosynthesis. Bacterial diversity has previously been investigated in TTX-containing host by polymerase chain reaction method [21,22]. However, only a small scale of gut bacteria was identified. Most of the reported TTX-producing bacteria are restricted to culturable bacteria in a laboratory [23,24] and the contribution of unculturable bacteria on TTX biosynthesis are underestimated.

To date, the secondary generation sequencing (SGS) is well developed to conduct bacterial community studies by 16S rRNA amplicon metagenomics [25]. Using high throughput sequencing technique, we conducted bacterial profiling studies in the gut environment of different puffer fish species with the most recent bioinformatic tools and pipelines, such as Quantitative Insights Into Microbial Ecology 2 (QIIME2) and DADA2 [26] for raw data processing and denoising, SATé-enabled phylogenetic placement (SEPP) for generating phylogenetic tree [27]. We also conducted the metagenome prediction by the newest Phylogenetic Investigation of Communities by Reconstruction of Unobserved States 2 (PICRUSt2) [28] on puffer fish gut microbiota data. To the best of our knowledge, it is the first study to profile the gut microbiota in puffer fish by an SGS approach, which will differentiate gut bacterial structures between toxic and non-toxic species and define the unique bacterial symbionts related to TTX biosynthesis. Our study will also identify the potential precursors in TTX biosynthesis through the metagenome prediction and elucidate their relations with the bacterial symbionts in the gut environment of puffer fish.

## 2. Results

### 2.1. Weight, Length and TTX Content in Puffer Fish Livers

**Figure 1:**
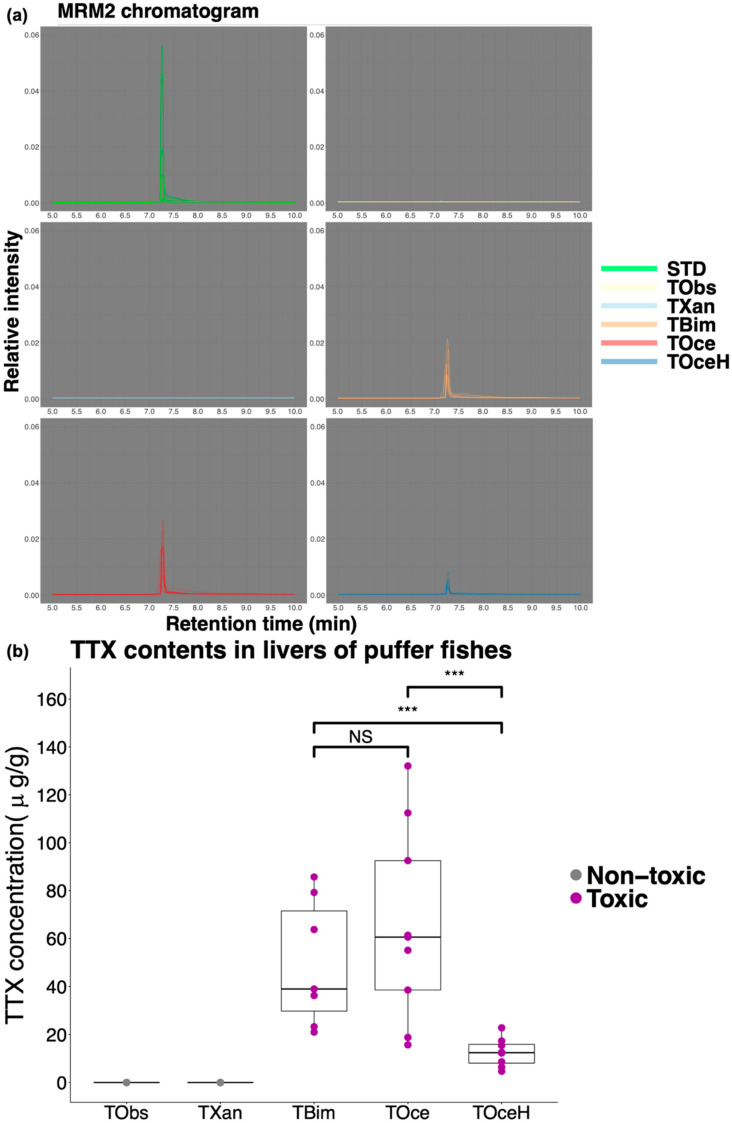
Puffer fish livers tetrodotoxin (TTX) contents detected by LC-MS/MS. (**a**) MRM2 (*m*/*z* 320.00 to *m*/*z* 161.80) chromatograms of TTX standards and samples. (**b**) TTX content boxplot. Wilcoxon rank-sum test was applied for significance testing of average TTX content between toxic species (*** *p*-value < 0.001; NS: Non-Significant). STD: standard; TObs: *T. obscurus*; TXan: *T. xanthopterus*; TBim: *T. bimaculatus*; TOce: *T. ocellatus*; TOceH: hybrid suspected *T. ocellatus*.

In this study, four wild species and one cultured specie were collected. The average weight and length of the puffer species were: *T. bimaculatus*: 125.8 ± 36.4 g and 16.2 ± 1.6 cm; *T. obscurus*: 326.8 ± 32.7 g and 23.6 ± 1.7 cm; *T. ocellatus*: 131.2 ± 33.2 g and 16.8 ± 2.8 cm; hybrid suspected *T. ocellatus*: 122.2 ± 40.2 g and 16.8 ± 2.9 cm; *T. xanthopterus*: 125.0 ± 37.6 g and 15.9 ± 2.1cm. TTX contents in the livers were detected by LC-MS/MS. MRM1 (*m*/*z* 320.00 to *m*/*z* 302.10) was applied for quantification and the calibration curve was constructed. The MRM1 chromatograms of samples and TTX standards were shown in Figure 1a, where all the peaks were aligned at time 7.2 min to 7.3 min. The average TTX contents in the liver samples were: *T. bimaculatus*: 49.75 ± 26.40 µg/g; *T. obscurus*: 0.00 µg/g; *T. ocellatus*: 65.25 ± 40.16 µg/g; hybrid suspected *T. ocellatus*: 12.53 ± 5.98 µg/g; *T. xanthopterus*: 0.00 µg/g. Boxplot showed the distribution of the TTX contents in different species (Figure 1b). Unquantifiable amount of TTX content was found in livers of *T. obscurus* and *T. xanthopterus*. Therefore, the cultured *T. obscures* and wild *T. xanthopterus* obtained in this study were grouped into the non-toxic species. *T*. *ocellatus*, suspected hybrid *T*. *ocellatus* species and *T*. *bimaculatus* were grouped into toxic species. In the toxic species, TTX contents in livers of *T*. ocellatus and *T*. bimaculatus were significantly higher than in the suspected *T*. ocellatus species (Wilcoxon rank-sum test, *p*-value < 0.001). Data of sex, weight, length, and liver TTX content of each sample was recorded in Supplementary File-Table S1. 

### 2.2. 16S rRNA Data of Puffer Fish Gut Microbiota

#### 2.2.1. 16S rRNA Raw Data Quality Control

In this study, 42 samples and 82 pair end sequence data were generated. Phred score Q30 indicated 99.9% accuracy was applied for assessing the raw data sequence data quality. An average of 95.4% ± 1.1% of Q30 among all sequence data were obtained in this study, indicating good sequencing quality (Supplementary File-Table S2). DADA2 [29] was applied for the sequence denoising. The number of outputted high quality sequence tags ranged from 86,322 to 198,501, and the average sequence utilization was 76.2% ± 11.4% (Supplementary File-Table S2).

#### 2.2.2. Bacterial Structure and Phylogenetic Diversity of Puffer Fish Gut Microbiota

**Figure 2:**
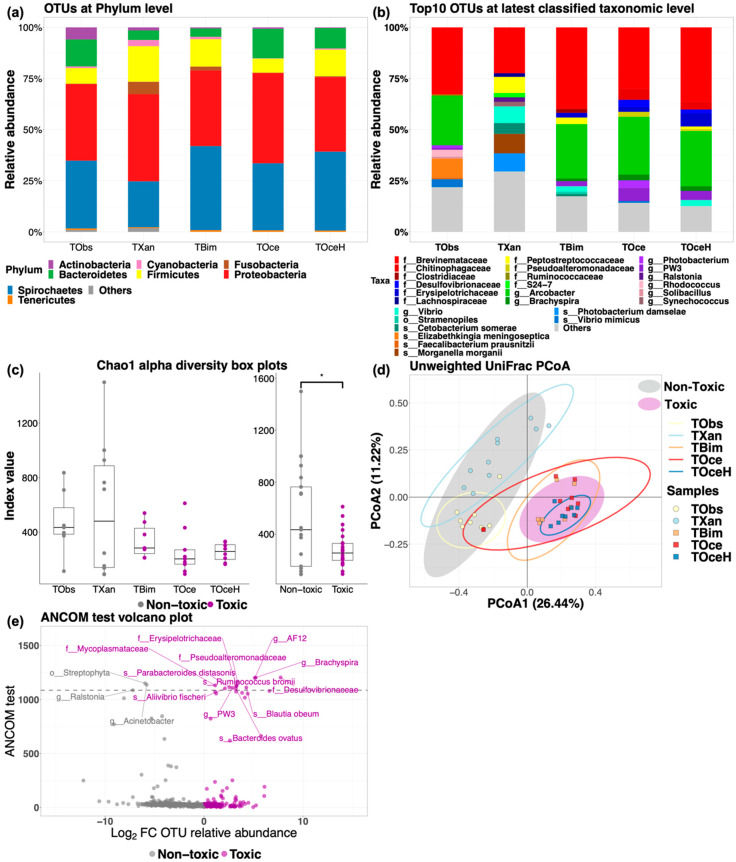
Puffer fish gut microbial structure and diversities. (**a**) Operational taxonomic units (OTUs) relative abundance at Phylum level. Phyla with relative abundance less than 1% were assigned to “Others”. (**b**) Top ten OTUs relative abundance at latest taxonomic levels. Low relative abundance OTUs outside the top ten were assigned to “Others”. (**c**) Chao1 alpha diversity boxplot. Significant testing was conducted by ANOVA among species, and Welch’s *T*-test between toxic group (*N* = 24) and nontoxic group (*N* = 18). * *p* < 0.05. (**d**) Unweight UniFrac principle coordinate analysis. Hollow ellipses and solid ellipses represented clustering of samples grouped by puffer species and toxicity respectively (95% confidence). The significance was tested by PERMANOVA (999 times permutation). (**e**) ANCOM test volcano plot. Dots with Log2FC > 0/Log2FC < 0 represents OTUs in gut microbiota of toxic/non-toxic puffer species. Dashed line separated OTUs that were significant distinct (above) and non-significant (below). TObs: *T. obscurus*; TXan: *T. xanthopterus*; TBim: *T. bimaculatus*; TOce: *T. ocellatus*; TOceH: hybrid suspected *T. ocellatus*.

In this study, 1207 unique operational taxonomic units (OTUs) were obtained and classified, where all the taxonomic classifications (Kingdom to Species) were recorded in Supplementary File-Table S3. At Kingdom level, 0.07% Archaea and 99.93% Bacteria were identified, indicating the dominant bacterial composition in the puffer fish gut microbiota. At phylum level, top phyla found in all species were Proteobacteria and Spirochaetes (*T. obscurus*: 37.39% ± 18.28%, 33.20% ± 27.13%; *T. xanthopterus*: 42.57% ± 28.37%, 22.50% ± 33.72%; *T. bimaculatus*: 36.96% ± 9.27%, 41.13% ± 13.48%; *T. ocellatus*: 44.15% ± 19.53%, 32.8% ± 14.57%; hybrid suspected *T. ocellatus*: 36.44% ± 7.91%, 38.62% ± 9.26%, Figure 2a). On the other hand, at the OTU level, taxa with top ten relative abundance of puffer fish gut microbiota were shown in Figure 2b. Family Brevinemataceae (phylum Spirochaetes) was mutually found in all the species with large dominance (*T. obscurus*: 32.79% ± 27.19; *T. xanthopterus*: 22.39 ± 33.69%; *T. bimaculatus*: 39.87 ± 13.68%; *T. ocellatus*: 30.02% ± 13.40%; hybrid suspected *T. ocellatus*: 36.36% ± 9.04%). In addition, another predominant bacterium was genus *Arcobacter*, which was found in all three wild species and the cultured species (*T. obscurus*: 24.08% ± 18.35%; *T. bimaculatus*: 26.57% ± 11.00%; *T. ocellatus*: 28.18% ± 18.03%; hybrid suspected *T. ocellatus*: 26.90% ± 10.40%).

Chao1 index value was applied to measure the bacterial richness of gut microbiota in different puffer species, box plot was applied to visualize the results (Figure 2c). The mean ± sd Chao1 index values of gut microbiota in different samples were 700.01 ± 677.48, 562.63 ± 490.24, 339.67 ± 129.38, 254.31 ± 167.30 and 252.91 ± 66.95 for *T. obscurus*, *T. xanthopterus*, *T. bimaculatus*, *T. ocellatus* and hybrid suspected *T. ocellatus* respectively. Wilcoxon rank sum test showed that Chao1 index value was significantly higher in the non-toxic group than in the toxic (*p*-value < 0.05).

Unweighted UniFrac dissimilarity matrix-based Principle coordinate analysis (PCoA) was applied for the phylogenetic diversity study of puffer fish gut microbiota (Figure 2d), PERMANOVA was applied for the significant testing. The testing results are recorded (Supplementary File-Table S4). PERMANOVA results showed that the cluster of toxic group samples (*N* = 24) was significantly different to the cluster of non-toxic group samples (*N* = 18, *p*-value < 0.001), indicating their dissimilarity of phylogenetic diversity. When samples were grouped by species in PERMANOVA pairwise comparison, significant results (*p*-value < 0.002) were found in the all the comparisons contained samples group of *T. xanthopterus* (*N* = 10) or *T. obscurus* (*N* = 8), indicating that gut bacteria compositions were unique in *T. xanthopterus* and *T. obscurus* in this study.

To distinguish the gut bacteria in toxic (*N* = 24) and non-toxic (*N* = 18) puffer species, ANCOM test was applied at the latest classified level of the data. ANCOM test results were recorded (Supplementary File-Table S5). Volcano plot showed all OTUs in the ANCOM test where the OTUs rejected by ANCOM test were labeled (Figure 2e). Families of Desulfovibrionaceae, Erysipelotrichaceae, Mycoplasmataceae, Pseudoalteromonadaceae; genera of *AF12 clone* (Rikenellaceae), *Brachyspira*, *PW3 clone* (Rikenellaceae); species of *Aliivibrio fischeri*, *Bacteroides ovatus*, *Parabacteroides distasonis* and *Ruminococcus bromii* were distinct in toxic puffer species, while order of Streptophyta; genera of *Ralstonia* and *Acinetobacter* were distinct in non-toxic puffer species.

### 2.3. Gut Bacteria Co-Occurrence Network

**Figure 3:**
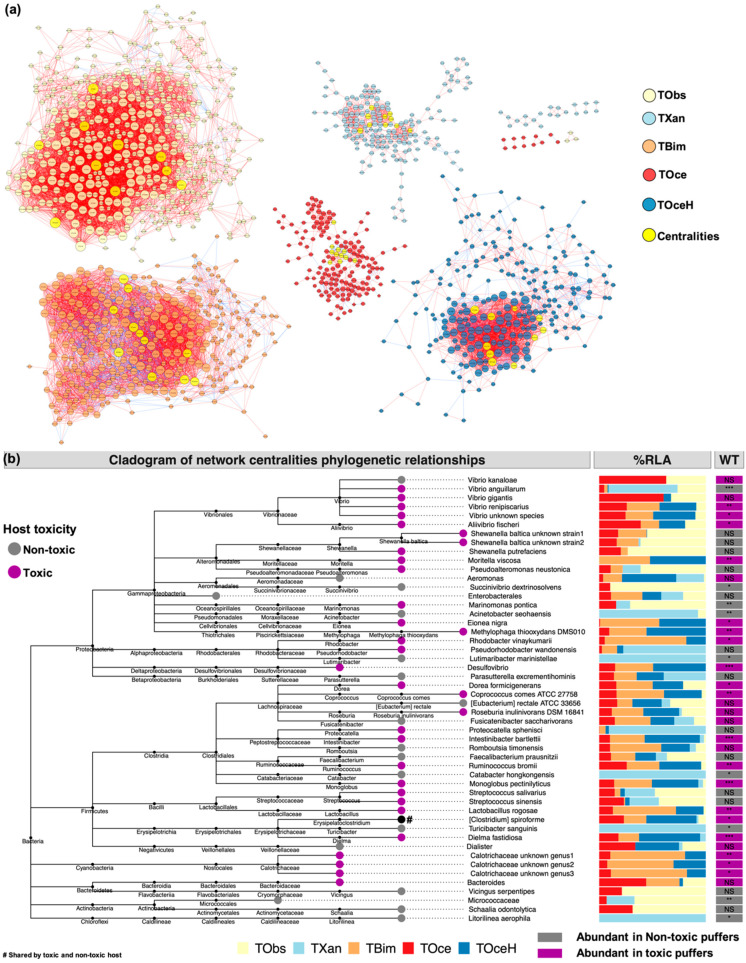
Puffer fish gut microbial co-occurrence network analysis. (**a**) Co-occurrence network at OTU level. Co-occurrence pairs with Spearman rho threshold >0.8 and *p*-value < 0.05 were shown. Edges showed the positive (red) and negative (blue) relationships between nodes. Network centralities in each puffer group were highlighted. (**b**) Cladogram showing common tree of network centralities and their percentage relative abundance (%RLA) in gut of puffer species. The relative abundance of the centralities in toxic (*N* = 24) and non-toxic puffer (*N* = 18) species were compared by Wilcoxon rank sum test (WT) (* *p* < 0.05, ** *p* < 0.01, *** *p* < 0.001; NS: Non-significant). TObs: *T. obscurus*; TXan: *T. xanthopterus*; TBim: *T. bimaculatus*; TOce: *T. ocellatus*; TOceH: hybrid suspected *T. ocellatus*.

Network analysis was conducted to study the significant co-occurrence pattern of bacteria in the gut environment of puffer fish. Significant correlation pairs (Spearman rho threshold >0.8, *p*-value < 0.05) were selected to construct the network (Figure 3a). All significant correlated pairs were recorded (Supplementary File-Table S6). A number of 51 unique network centralities were determined by incorporating the rank of their network features (Supplementary File-Table S7). The representative sequence of the centralities and their BLAST results were recorded in Supplementary File-Tables S8 and S9. Cladogram showing the common lineage of the centralities were constructed (Figure 3b). In the BLAST result, 50 out of 51 centralities were successfully classified. The centralities in all puffer groups were identified within phyla of Proteobacteria, Firmicutes, Cyanobacteria, Bacteroidetes, Actinobacteria, and Chloroflexi. Among them, the majority of centralities belonged to phyla Proteobacteria (23/50) and Firmicutes (19/50). In Proteobacteria lineage, *Vibrio anguillarum*, *V. gigantis*, *V. renipiscarius*, *Aliivibrio fischeri* and an unknown *Vibrio* species were found to be network centralities of toxic puffer species, where the latter three *Vibrio* species were significantly abundant (*p*-value < 0.05) in toxic puffer species than in the non-toxic. Additionally, centralities belonging to Alteromonadales (two unknown *Shewanella baltica* strains, *S. putrefaciens*, *Moritella viscosa* and *Pseudoalteromonas neustonica*) were all found in the toxic groups, where the relative abundance of *M. viscosa* was found significantly higher (*p*-value < 0.01) in toxic puffers. Meanwhile, some other centralities belonging to phylum Proteobacteria, such as *Eionea nigra*, *Methylophaga thiooxydans* DMS010 strain, *Rhodobacter vinaykumarii* and genus *Desulfovibrio* were uniquely found in toxic puffer species with significantly higher relative abundance (*p*-value < 0.05). On the contrary, centralities in non-toxic host representing *Acinetobacter seohaensis* and *Lutimaribacter marinistellae* within phylum Proteobacteria were found significantly abundant (*p*-value < 0.05) in non-toxic puffer species.

On the other hand, centralities belonging to phylum Firmicutes were evenly distributed in toxic species and in the non-toxic species (Figure 3b). One clade with more than half of the centralities in Firmicutes lineage was class Clostridia that *Dorea formicigenerans*, *Coprococcus comes* ATCC 27758 strain, *Intestinibacter bartlettii*, *Ruminococcus bromii* and *Monoglobus pectinilyticus* were found significantly abundant in toxic puffers, while *Catabacter hongkongensis* was uniquely found in non-toxic *T. xanthopterus*. In particular, another clade of centralities belonging to order Lactobacillales (*Streptococcus salivarius*, *S. sinensis* and *Lactobacillus rogosae*) were intensively found in the toxic puffer species, though only *L. rogosae* was found significantly higher (*p*-value < 0.01) in toxic puffers. Other centralities belonging to Firmicutes lineage, such as *Dielma fastidiosa* was found dramatically abundant in toxic species (*p*-value < 0.001) while *Turicibacter sanguinis* were again found in *T. xanthopterus* exclusively.

One special clade belonging to Cyanobacteria lineage showed that all the centralities in this clade (three unknown Calotrichaceae) were found in the toxic group along with their significantly higher (*p*-value < 0.05) relative abundance in the toxic species than the non-toxic one. On the other hand, two non-toxic group centralities in different lineage (Micrococcaceae belonging to Actinobacteria, and *Litoriline aaerophile* belonging to Chloroflexi) were found significantly higher in non-toxic puffers (*p*-value < 0.05).

### 2.4. Key Metabolic Pathway Prediction and Distinction

**Figure 4:**
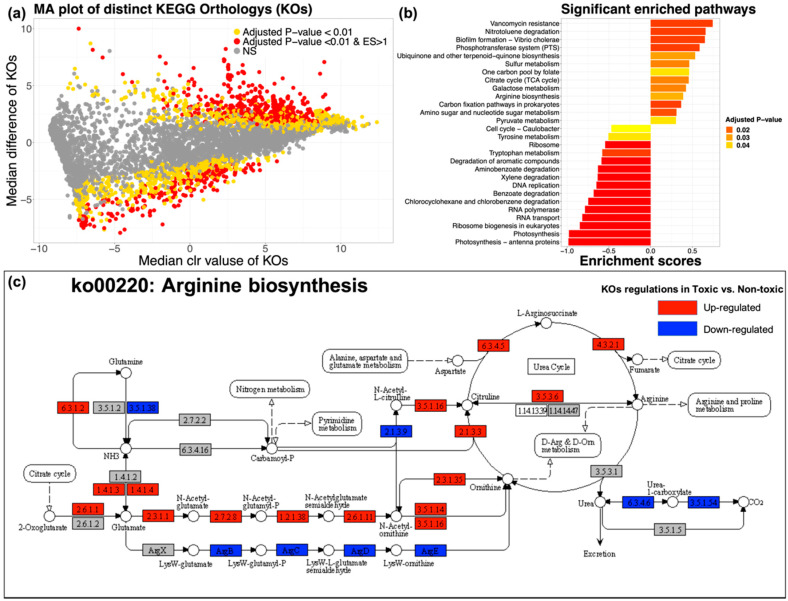
PICRUSt2 KEGG Orthology (KO) prediction, distinction and gene set enrichment results. (**a**) MA plot showing significant distinct KOs determined by ALDEx2 algorithm between toxic group (*N* = 24) and non-toxic group (*N* = 18). KOs with BH adjusted *p*-value < 0.01 and effect size >1 (distinct in toxic group) or effect size <−1 (distinct in non-toxic) were considered to be significant distinct. Only KOs with effect size > 1 and BH adjusted *p*-value < 0.01 were applied for the subsequent gene set enrichment analysis. (**b**) Significant enriched KEGG pathway bar plot. KEGG pathways with adjusted *p*-value < 0.05 in gene set enrichment analysis (GSEA) were considered as significantly enriched (Enrichment scores >0: enriched in toxic group; <0: enriched in non-toxic group). (**c**) Arginine biosynthesis. Significantly up/down (red/blue) regulated KOs and non-significant (gray) KOs revealed from ALDEx2 analysis in the pathway were shown. 1.2.1.38: N-acetyl-gamma-glutamyl-phosphate reductase; 1.4.1.3: glutamate dehydrogenase; 1.4.1.4: glutamate dehydrogenase; 2.1.3.3: ornithine carbamoyltransferase; 2.1.3.9: N-acetylornithine carbamoyltransferase; 2.3.1.1: N-acetylglutamate synthase; 2.3.1.35: glutamate N-acetyltransferase; 2.6.1.1: aspartate aminotransferase; 2.6.1.11: acetylornithine aminotransferase; 2.7.2.8: N-acetylglutamate 5-phosphotransferase; 3.5.1.14: aminoacylase; 3.5.1.16: acetylornithine deacetylase; 3.5.1.38: glutamin-(asparagin-)ase; 3.5.1.54: allophanate hydrolase; 3.5.3.6: arginine deiminase; 4.3.2.1: argininosuccinate lyase; 6.3.1.2: glutamine synthetase; 6.3.4.5: argininosuccinate synthase; 6.3.4.6: urea carboxylase; ArgB: LysW-gamma-l-alpha-aminoadipate; ArgC: LysW-gamma-l-alpha-aminoadipyl-6-phosphate; ArgD: LysW-gamma-l-lysine; ArgE: (amino group carrier protein)-lysine.

PICRUSt2 [28] was adopted to conduct the KEGG Orthology (KO) prediction. All the predicated KOs were recorded (Supplementary File-Table S10). R package ALDEx2 was applied to identify the distinct KOs in the comparison between toxic and non-toxic groups. The ALDEx2 test results were recorded in Supplementary File-Table S11. KOs with effect size threshold >1 and/or BH adjusted *p*-value < 0.01 were considered to be distinct KOs. MA plot was applied to show the significant distinct KOs in ALDEx2 (Figure 4a). The significant distinct KOs were loaded into R package *clusterprofile* for GSEA with default setting. All significant enriched KEGG pathways (BH adjusted *p*-value < 0.05) from GSEA were recorded in Supplementary File-Table S12. Selected significant enriched pathways relating to chemical biosynthesis and metabolisms were shown in Figure 4b. In the results, arginine biosynthesis (ko00220) was found significantly enriched in toxic group. Arginine biosynthesis pathway was revised from KO database by R package *pathview* and detailed KO changes inside were further investigated (Figure 4c). In arginine biosynthesis, a clear pathway of the bio-reaction from glutamate to arginine was found significantly up-regulated in the comparison between the toxic and non-toxic groups. Significant up-regulations (BH adjusted *p*-value < 0.01) of N-acetylglutamate synthase (2.3.1.1), acetylglutamate kinase (2.7.2.8), N-acetyl-gamma-glutamyl-phosphate reductase (1.2.1.38), acetylornithine aminotransferase (2.6.1.11), aminoacylase (3.5.14) and acetylornithine deacetylase (3.5.1.16) indicated that glutamate was more effectively transferred to omithine in the gut microbiota of toxic puffers. Subsequently, the significant up-regulations (BH adjusted *p*-value < 0.01) of ornithine carbamoyltransferase (2.1.3.3), argininosuccinate synthase (6.3.4.5) and argininosuccinate lyase (4.3.2.1) showed that omithine was significantly transferred to citrulline, argininosuccinate, and then to arginine in the toxic group. In addition to arginine biosynthesis, pathway representing *Vibrio cholerae* biofilm formation was found significantly enriched in the gut environment of toxic puffers, indicating the high activity of *Vibrio* species in this environment.

## 3. Discussion

### 3.1. Comparison of Toxicity and Gut Microbiota between Toxic and Non-Toxic Puffer Species

In this study, the living environments of both the cultured and wild puffer species were similar. The body weight and length of culture *T*. *obscurus* were significantly larger than the wild species, indicating the food supply for the culture species was sufficient and stable than from the wild environment. Not surprisingly, the TTX content in *T*. *obscurus* was non-detectable, which was in line with the previous study that toxic puffer fish would become non-toxic after culturing [30]. On the other hand, referring to non-toxic *T*. *xanthopterus* reported previously [31], the findings of wild *T*. *xanthopterus* with non-detectable TTX content in liver was reasonable. The wild but non-toxic puffer species provides valuable hints for the origin of TTX. In our study, microbial richness in *T*. *xanthopterus* and *T. obscurus* was significantly higher than in the toxic species. In addition, UniFrac-based PCoA and PERMANOVA test also suggested the significant difference of microbial structure between toxic and non-toxic puffer fish gut microbiota. In the case of sharing the same living environment, the difference of gut microbiota between *T*. *xanthopterus* and other wild toxic puffers may be contributed by their diet, which could alter entire fish gut microbial structure [18]. Though inherent factors of puffer fish may influence the bacteria composition, recent study on different fish diet altering the bile acid composition as well as the gut bacteria composition in grass grap indicated the importance of diet in shaping gut microbiota [32]. Therefore, it was suspected that *T*. *xanthopterus* may has a different food preference comparing to the other wild puffer in this study, which eventually formed a different gut microbiota structure.

In this study, we found that the most dominant phylum in all the species was Proteobacteria, which was in line with the previous study on the culturable bacterial study on *T*. *obscures* [21]. With 16S rRNA metagenomic study, we extended the knowledge of predominant bacteria in puffer fish gut. We found that that a considerable amount of Spirochaetes, Firmicutes, and Bacteroidetes (Figure 2a) also constructed the gut microbiota composition in toxic, non-toxic, wild-caught, and farm-raised puffer fish. Exact phylogenetic classifications of most of the OTUs were unable to be obtained due to the deficiency of the taxonomic database. Nevertheless, a number of OTUs classified at a species level were significantly differentiated in the ANCOM test (Figure 2e). Among the results, *Aliivibrio fischeri* was found significantly abundant in the toxic species. Besides, *Vibrio* species were also massively found as network centralities in the gut environment of the toxic species, indicating that *Vibrio species* and *Aliivibrio species* may play a more crucial role in the bacterial community in the toxic species than in the non-toxic one. Resent study showed that bacterial symbionts were adversely affected by the type VI secretion system of *V. cholera* [33], which could explain the significantly lower of microbial richness in toxic gut environment functionally dominate by *Vibrio species.* Unique gut microbiota was possibly shaped by unique food preference of toxic puffer species, which might subsequently create the essential conditions for TTX-biosynthesis. On the other hand, recent study showed that planocerid flatworm sequences were frequently detected in toxic puffer *Chelonodon patoca*, suggesting that toxic puffer fish accumulate TTX by preying planocerid flatworm eggs [34]. However, neither the producing mechanism of TTX nor the bacteria composition in the digestion system of the flatworm were investigated. It would be possible that planocerid flatworm or other TTX holder such as octopus shared similar bacterial keystones in the digestive systems for TTX biosynthesis. Our study offers references of gut microbiota in toxic and non-toxic puffer fish for further studies on digestive system of other TTX holders.

### 3.2. Vibrio spp. and Calotrichaceae (Cyanobacteria) TTX-Producing Symbionts

In this study, bacteria in the gut environment of the toxic species were found consistent in both of the ANCOM test showing distinct abundance and the network centralities showing the functional keystones in the bacterial community [35]. For instance, *Ruminococcus bromii* and *Aliivibrio fischeri* were found both distinct abundant (ANCOM test) and as network centralities in toxic group, suggesting their potential relationships or participation in TTX biosynthesis. The role of *Ruminococcus* species in the gut environment of puffer fish was unclear. However, the detection of *Ruminococcus* species found in the gut of *Siganus canaliculatus* fed by non-starch polysaccharide [36] suggested the role of *Ruminococcus* species in cellulose degradation of fish gut. Furthermore, rich and diverse cellulose-degrading enzymes in *Ruminococcus* species transferring polysaccharides into nutrient source for other bacterial symbionts and the host revealed their key role in the gut environment [37].

Similar to the findings of *R. bromii*, *Aliivibrio fischeri* was also found as network centrality in the gut environment of toxic puffers with significantly higher abundance comparing to the non-toxic group. *Aliivibrio fischeri* was a TTX-producing bacterium found in *Atergatis floridus* [38]. The relatively predominance of it in the toxic puffer fish guts directly implied its close relationship with TTX biosynthesis. Additionally, centralities representing *Vibrio* species were mostly found in toxic puffers (Figure 3b). Among them, TTX-producing ability of *V. gigantis*, *V. renipiscarius* were unknown. However, they were originally isolated from marine species [39,40] and were believed to colonized in puffer fish gut thorough marine environment. Secondary metabolites were frequently found in the symbiotic bacteria community composed of *Vibrio* [41]. Though *Vibrio* was the most frequent genus reported as TTX-producing bacteria [42], no *Vibrio* strain was successfully isolated for continuously TTX production. With respect to this fact, symbionts composed of *Vibrio* species or potential triggers were suspected for TTX biosynthesis.

In addition to *Vibrio* species, Calotrichaceae belonging to Cyanobacteria lineage was found centered in the microbial co-occurrence network of the toxic samples (Figure 3a,b). Little research reported the presence of Cyanobacteria in the gut environment of puffer fish. However, as the major inhabitant of the marine environment, Cyanobacteria may passively get into puffer fish gut. Previous study showed that Cyanobacteria served as reservoir of *V. cholerae* [43]. Therefore, *Vibrio* and Cyanobacteria may form a symbiotic relationship in the gut environment of toxic puffer fish in this study. Cyanobacteria were known to be responsible for saxitoxin (STX) biosynthesis, whose structure shares a similar guanidino group with TTX. *Fugu pardalis* was previously found to host both TTX and STX [44], indicating their nonexclusive relationship. The biosynthesis of saxitoxin was achieved by a massive of gene clusters in Cyanobacteria [45]. In the case of TTX, the exact gene or gene clusters coding for TTX biosynthesis were unknown. The significant co-occurrence of *Vibrio* and Cyanobacteria in the gut environment of toxic puffer fish possibly determine the TTX biosynthesis. However, whether TTX and STX are inter-correlated such as sharing similar precursors or intermediates during their biosynthesis is unknown and required further studies.

On the other hand, though *Vibrio* species were also found in the non-toxic puffers, only one of them were defined as network centralities that *Vibrio* species were considered to be less functionally important in the gut environment of non-toxic species than in the toxic. In addition to *Vibrio* and Cyanobacteria, *Shewanella baltica* and *Pseudoalteromonas neustonica* were found as network centralities in the toxic puffers in this study, though their relevance were not significant in comparison to the non-toxic group. Genus *Shewanella* and genus *Pseudoalteromonas* of unknown species were identified as TTX-producing bacteria in *Nassarius semiplicatus* and *Hubrechtella juliae* respectively [46,47]. Therefore, *Shewanella baltica* and *Pseudoalteromonas neustonica* identified in this study may also contribute to TTX biosynthesis. However, bacterial stain level identification though short amplicon sequencing is difficult that further investigations are required to confirm the exact strain of *Shewanella* and *Pseudoalteromonas* in this study.

Factors shaping different gut microbiota between toxic and non-toxic puffers were diversified. The compositional changes of bacteria symbionts in puffer fish gut was possible to determine the presence of TTX. The exclusive finding of *Moritella* sp., *Eionea* sp., *Methylophaga* sp., *Rhodobacter* sp., *Desulfovibrio* sp. and Calotrichaceae in toxic puffers and their importance as network centralities strongly suggested that gut environment of toxic samples catered their growth as symbionts, which also favored TTX biosynthesis as secondary metabolites. Our study defines the gut bacteria symbionts with the major components of *Vibrio* sp. and Cyanobacteria. *In vitro* co-culture of the *Vibrio* spp. and cyanobacteria can further validate the TTX-biosynthesis in the symbionts.

### 3.3. Metabolic Pathways Relating to TTX Biosynthesis

Arginine has been proposed to be the precursor in the TTX biosynthesis, serving as amidino donor of the guanidinium moiety in TTX [8], however, has yet to be proved. In this study, the PICRUSt2 genome prediction and GSEA result predicted that arginine biosynthesis was significantly more activated in the gut microbiota of toxic puffers than in the non-toxic, which supported the previous proposal. Arginine biosynthesis from glutamate was well documented in different bacteria [48], as well as in *Vibrio* and Cyanobacteria [49,50]. The pattern of arginine biosynthesis in *Vibrio* and Cyanobacteria were identical that glutamate was catalyzed to omithine, citrulline, argininosuccinate, and finally to arginine, where all genes coding for the catalyzations were significantly enriched in the gut microbiota of toxic puffers in our study. Although other gut bacteria in puffer fish may also contribute to the arginine biosynthesis, the identical biosynthetic pattern and the key role of *Vibrio* and Cyanobacteria in the gut microbiota symbiont at least indicated their participation of arginine biosynthesis in the gut environment of toxic puffers. Nonribosomal peptide synthetase (NRPS) richly found in *Vibrio* and Cyanobacteria [51,52] were suspected to utilize arginine for the incorporation of guanidinium moiety of TTX [8]. However, the presence of NRPS and its relationship to TTX biosynthesis are required to be investigated in future studies.

In addition to the findings of arginine biosynthesis, the pathway representing *Vibrio cholerae* biofilm formation was found to be significantly enriched in the toxic puffer fish gut environment, which further emphasized the importance of *Vibrio* in both arginine biosynthesis as well as the TTX biosynthesis in the gut environment of puffer fish. Although the strain level of *Vibrio* was not commonly identified, *Vibrio* genus was frequently found in the gut environment of marine fish [18]. *Vibrio* was known as opportunistic bacteria [53]. Recent study showed that the antibiotic-induced reduction of bacterial richness and diversity in fish gut increased the dominance of opportunistic *Vibrio* in the gut environment [54]. In line with our study, microbial richness was found significant lower in the toxic puffer gut environment, where the *Vibrio* was found centered at the bacteria co-occurrence network with significantly high relative abundance than that in the non-toxic gut environment. Coupling with the finding of the significantly enriched metabolic pathway relating to *Vibrio cholerae* biofilm formation in the toxic group, we suspected that opportunistic *Vibrio* may dominant the toxic puffer fish gut environment and possibly contributed to TTX biosynthesis. The expression of opportunistic *Vibrio* was found different *in vitro* and *in vivo* [18], which could explain the low TTX detection of *Vibrio in vitro* [17]. However, specific unknown or unidentified *Vibrio* strain may be responsible for the TTX production and awaited further confirmation.

## 4. Materials and Methods

### 4.1. Puffer Fish Sample Collections

All puffer fish samples were collected in the Pearl River Estuary region. The three wild species, *Takifugu ocellatus*, *T. bimaculatus*, *T. xanthopterus* were collected from the north of Qi Ao Island located in Pearl River, Zhuhai city (113.65° E, 22.50° N, Figure S1). Some *T. ocellatus* samples with abnormal stripes (Figure S2) were suspected to be a hybrid species which were assigned to a unique comparison group in this study. One cultured species *T. obscurus* was collected from a fish farm at the coastal area of Zhongshan city (113.55° E, 22.55° N, Figure S1). The water source of the of the fish farm was directly from the Pearl River. After sample collection, the length, weight, and sex of each sample were recorded (Supplementary File-Table S1). The removal of fish gut from each sample was conducted in a sterile safety cabinet and its gut content was collected in a 2.0 mL sterilized centrifuge tube and stored in −80 °C before analysis, whereas the livers of the samples were stored in −20 °C before analysis.

### 4.2. TTX Extraction

The extraction of TTX from the liver samples followed the previous method with some modifications [55]. Briefly, 10.0 g of homogenized liver sample was weighted in a 50.0 mL centrifuge tube. 1:2.5 *w*/*v* 0.1% acetic acid was added. The mixture was heated in a boiling water bath for 10 min and then cooled to room temperature before centrifugation at 4500 rpm for 5 min. The supernatant was separated out from the tube and was added into another tube containing 1:1 *v*/*v* hexane which would undergo vibration in a shaker for 15 min. Subsequently, hexane was discarded, and the extract was transferred into a 50.0 mL volumetric flask and filled up with 0.1% acetic acid. Finally, each sample solution was passed though the 0.2 μm polytetrafluoroethylene (PTFE) syringe filter and stored into the 1.5 mL vial and stored at 4 °C before the Ultra High Pressure Liquid Chromatography tandem Mass Spectroscopy (UHPLC-MS/MS) analysis.

### 4.3. UHPLC-MS/MS Conditioning and TTX Detection

TTX standard (purity ≥ 99%) was purchased from a commercial company (Shanghai Aladdin Bio-Chem Technology Company Limited, Shanghai, China). Working solutions with TTX concentration ranged from 0.1 μg/g to 100 μg/g were prepared for standardization.

TTX detection by UHPLC-MS/MS was optimized in our lab. Agilent (Agilent Technology, Inc, Santa Clara, CA, USA) 1290 UHPLC system and Agilent (Agilent Technology, Inc, Santa Clara, CA, USA) 6460 triple quadrupole mass spectroscopy system were applied for TTX identification and quantification. The column for separation was Agilent Hilic Plus (RRHD 1.8 μm, 2.1 × 100 mm, Agilent Technology, Inc, Santa Clara, CA, USA). The mobile phase was 0.1% acetic acid in water (A) and acetonitrile (B). The elution program was: 0–6.0 min: A:B = 1:99, 6.0–6.2 min: A:B = 95:5, 6.2–8.2 min: A:B = 95:5, 8.2–8.5 min: A:B = 90:10, 8.5–10.0 min: A:B = 90:10, 10.0–10.5 min: A:B = 50:50. Post time = 5 min. The mobile phase flow rate was 0.4 mL/min and the column temperature was 25 °C. The injection volume was 1 µL.

For the mass spectroscopy, electrospray Ionization positive mode (+ESI) was applied as the ionization source. The scan type was multiple reaction monitoring (MRM). MRM1 was set at collision induced dissociation energy (CID) = 25 eV, segment was 320.0 *m*/*z* to 302.1 *m*/*z*. MRM2 was set at CID = 35 eV, segment was 320.0 *m*/*z* to 161.8 *m*/*z*. The source parameters of the mass spectroscopy were: gas temperature = 350 °C, gas flow = 8 L/min, nebulizer = 45 psi, sheath gas temperature = 400 °C, sheath gas flow = 11 L/min, capillary voltage = 4000 V. The identification of TTX was based on the MRM1 and MRM2. The quantification of TTX was based on the MRM1.

### 4.4. 16S rRNA DNA Extraction and Library Preparation

The total DNA in each gut sample was extracted using CTAB/SDS method. DNA concentration and purity were monitored on 1% agarose gels. All PCR reaction was carried out with Phusion^®^ High-Fidelity PCR Master Mix (New England BioLabs, Ipswich, MA, USA). The primer pair for 16S V3-V4 regions was 314F: CCTAYGGGRBGCASCAG; 806R: GGACTACNNGGGTATCTAAT. PCR productions were loaded on 2% agarose gel and length with 400–450bp was chosen for further experiments. The PCR product purification was conducted by Qiagen Gel Extraction Kit (Qiagen, Hilden, Germany). Sequencing libraries was generated using NEBNext^®^ Ultra^TM^ DNA library Pre-Kit for Illumina^®^ (New England BioLabs, Ipswich, MA, USA) with the addition of index codes. The quality control of the libraries was conducted by Agilent Bioanalyzer 2100 (Agilent Technology, Inc, Santa Clara, CA, USA). The DNA sequencing was conducted by Illumina Hiseq 2500 platform by Novogene company (Beijing, China).

### 4.5. Statistical Analysis

#### 4.5.1. TTX Content

The TTX concentration in the liver samples was calculated by the calibration curve prepared by the TTX standard solutions. The chromatogram information (retention time, intensity) of MRM were obtained by Masshunter software (Agilent Technology, Inc, Santa Clara, CA, USA). Box plot was applied to show the distribution of TTX content in the liver of different puffer species. Wilcoxon rank-sum test was applied to test the difference of TTX content between puffer species.

#### 4.5.2. 16S rRNA Raw Data Processing

16S rRNA raw sequence data were generated by Illumina Hiseq 2500. Barcodes and primers were truncated and the data were imported into Quantitative Insights Into Microbial Ecology2 (QIIME2, version 2019.7) [26] platform for downstream analysis. *DADA2* [29] algorithm was applied for the sequences denoising, generating representative sequence and OTU table. SATé-enabled phylogenetic placement (SEPP) algorithm [27] in *fragment-insertion* plugin of QIIME2 platform was applied to generate a reliable phylogenetic tree against the 99% GreenGene [56] database (Version: 13.8). Taxonomic classification was conducted by QIIME2 plugin *feature classifier*. All the sequence data were rarefied to 86,322 sequences per sample for the diversity analysis.

#### 4.5.3. OTU Relative Abundance

Total sum scaling was applied to the OTU table to calculate the relative abundance of OTU in each gut sample. OTUs relative abundances with taxonomic classifications were shown with stacked bar chart. 

#### 4.5.4. Alpha Diversity Analysis

Chao1 index values of each gut sample were calculated [57]. Boxplot was applied to visualize the results. Gut samples were grouped by the toxicity of the puffer species and the significant difference of Chao1 index values between toxic and non-toxic groups were tested by Welch’s *T*-test.

#### 4.5.5. Beta Diversity Analysis

Unweighted Unifrac [58] dissimilarity matrix of gut samples was constructed with the input of OTU table and phylogenetic tree resulted from SEPP. Principle coordinate analysis (PCoA) was conducted on the matrix and the score plot was applied to visualize the results [59]. Permutational multivariate analysis of variance (PERMANOVA) [60,61] was applied for the testing of significant clustering between sample groups.

#### 4.5.6. Distinct OTUs Identification

Distinct bacteria from the comparison between gut microbiota of toxic and non-toxic puffer species were revealed by Analysis of composition of microbiomes (ANCOM) [62]. OTUs rejected by the ANCOM test was considered to be significantly distinct. Volcano plot was applied to visualize the results.

#### 4.5.7. OTU Co-Occurrence Network Analysis

Samples were grouped by the species and the Spearman coefficient correlation matrix was constructed for each group. Selected pairs of OTUs (Spearman rho threshold = 0.8, *p* < 0.05) were loaded into Cytoscape software version 3.7.2 (The Cytoscape Consortium, New York, NY, USA) for network analysis. The correlations between OTUs and host body weight, body length, and liver TTX content in each group were also involved. Network Analyzer [63] plugin in Cytoscape software was applied for the calculation of network features (Betweenness Centrality, Closeness Centrality, Neighbourhood Connectivity, Degree, and Topological Coefficient). Network centralities were determined by the incorporation of network features. Detail taxonomic classification of the network centralities were determined by blasting their representative sequence against the NCBI 16S rRNA database by NCBI online BLAST tool (blast.ncbi.nlm.nih.gov/Blast.cgi). The relative abundances of the centralities were rescaled to 100% and shown with stacked bar chart. Significant differences of centralities between toxic and non-toxic species were tested by Wilcoxon rank sum test.

#### 4.5.8. KEGG Orthology (KO) Prediction and Distinction

Phylogenetic Investigation of Communities by Reconstruction of Unobserved States2 (PICRUSt2) was adopted for KO prediction from 16S rRNA data [28]. SEPP was applied for fragment insertion of representative sequence against the reference sequence and reference tree constructed from Integrated Microbial Genomes and Microbiomes [64] in PICRUSt2. The generated tree was then complemented with the OTU table resulted from DADA2 for hidden-state prediction (hsp) [65] using maximum-parsimony method, generating predicted KOs. ALDEx2 [66] algorithm was applied to calculate the effect size of KOs and conducted the significant testing in the comparison between toxic group and non-toxic group samples. KOs with effect size threshold >1 and/or Benjamin-Hochberg (BH) adjusted *p*-value < 0.01 (Wilcoxon rank sum test) were identified as distinct KOs. MA plot was applied to visualize the results.

#### 4.5.9. Pathway Studies

Gene set enrichment analysis (GSEA) [67] was applied to determine the significant enriched metabolic pathway in KEGG Orthology database with the input of distinct KOs (effect size threshold >1 and/or BH adjusted *p*-value < 0.01). Resulted metabolic pathways from the GSEA with BH adjusted *p*-value < 0.05 were considered as significant enriched. Bar chart was applied to show the significant enriched pathways. Interested significant enriched metabolic pathways were predicted from KEGG Orthology database.

#### 4.5.10. Bioinformatic Software and R Packages

QIIME2 *dada2* plugin and *fragment*-*insertion* plugin were applied for the DADA2 and SEPP workflow. QIIME2 *diversity* plugin was applied for calculating the Chao1 index value, generating the Unweighted Unifrac dissimilarity matrix and conducting principle coordinate analysis and PERMANOVA test. QIIME2 *feature classifier* plugin was applied to conduct the taxonomic classification. QIIME2 *composition* plugin was applied to conduct the ANCOM test.

R (version 3.6.1) was used for data visualization and some of the significant testing in this study. R *ggplot2* package was applied to construct the chromatogram, boxplot, stacked bar chart, score plot, volcano plot, and MA plot. R *stat* package was applied to calculate the average values, fold change (FC) values of the OTU relative abundance, and to conduct the Welch’s *T*-test and Wilcoxon rank sum test. R *ggtree* package [68] was applied to construct the cladogram. R *ALDEx2* package [66] was applied to determine the significant distinct KOs. R *clusterprofile* package and *gseKEGG* algorithm [69] was applied to conduct GSEA adopting the KEGG Orthology database. R *pathview* package [70] were applied to visualize the interested KEGG pathways.

#### 4.5.11. Data Availability

The sequence data in this study has submitted to NCBI database with reference to SRA submission SUB7371895.

## 5. Conclusions

Using 16S rRNA amplicon metagenomic study on the gut content of both toxic and non-toxic puffer fish, a significant difference in bacterial structures was identified between the toxic and non-toxic samples. In the co-occurrence network, *Vibrio* and Cyanobacteria were defined as key symbionts in the gut environment of toxic species, possibly contributing to TTX biosynthesis. PICRUSt2 and GSEA results predicted that arginine biosynthesis was activated in the gut environment of toxic puffers. Our study supports the hypothesis of TTX biosynthesis inside the hosts by their gut bacterial symbionts using arginine as a precursor.

## Supplementary Materials

The following are available online at https://www.mdpi.com/1660-3397/18/5/278/s1, Table S1: Weight, length, sex and liver TTX content of different puffer fish species, Table S2: %Q30 and summary of DADA2 sequence data quality control, Table S3: OTU table at different taxonomic levels, Table S4: Permanova test results, Table S5: ANCOM test result (Toxic vs. non-toxic), Table S6: Significant correlation pairs, Table S7: Parameters in network analysis and their rank scores, Table S8: Representative sequences of network centralities, Table S9: NCBI BLAST results of network centralities, Table S10: PICRUSt2 KO prediction results, Table S11: ALDEx2 results, Table S12: Significant enriched KEGG pathways in gene set enrichment results, Figure S1: Map of sampling locations, Figure S2: Puffer species in this study.
