# Supplementary figures and images for: Puffer Fish Gut Microbiota Studies Revealed Unique Bacterial Co-Occurrence Patterns and New Insights on Tetrodotoxin Producers
Source: Mar Drugs. 2020 May 25;18(5):278. doi: 10.3390/md18050278 (PMC7281374; doi:10.3390/md18050278)

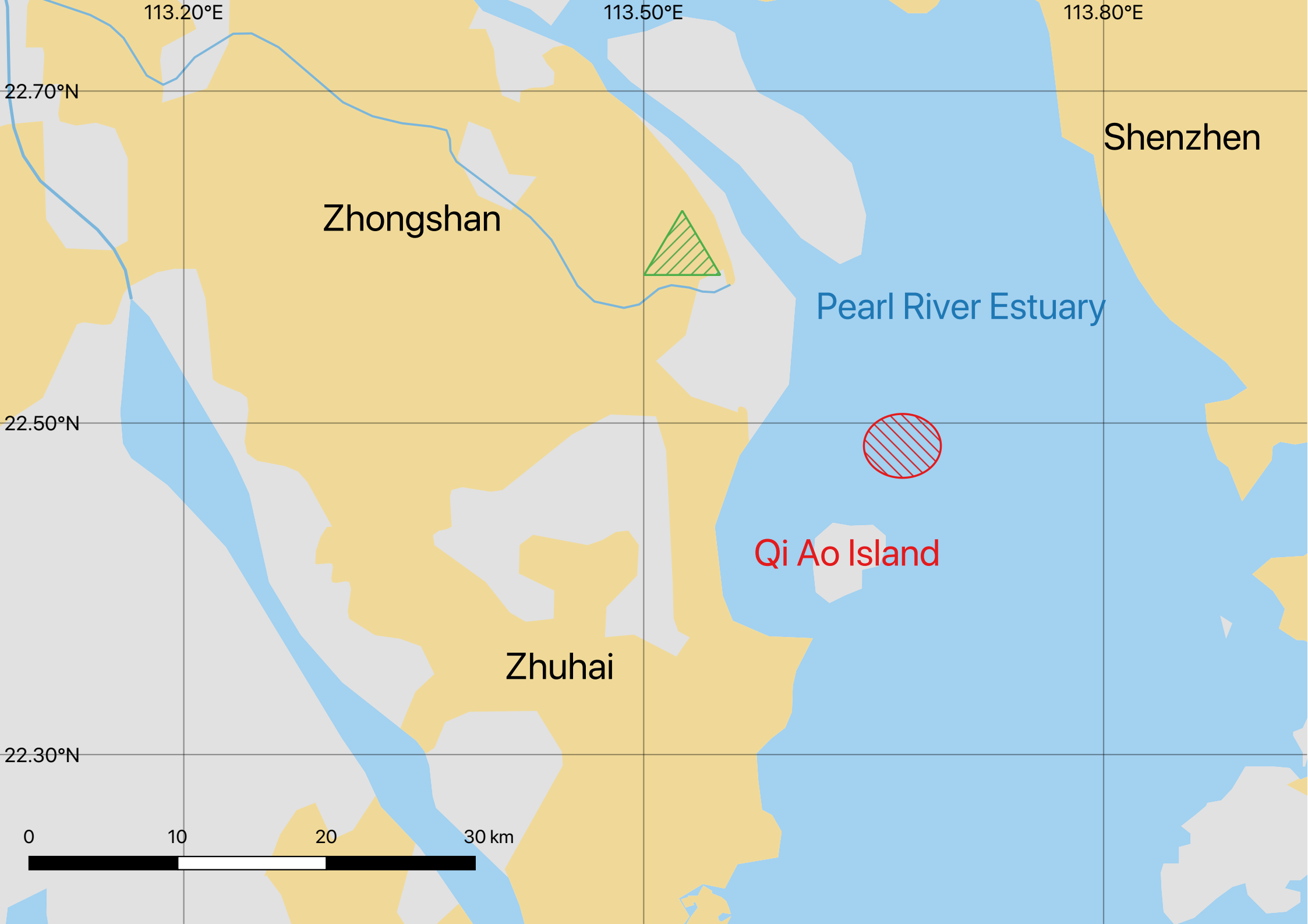

Supplement: Supplementary file 1 [file marinedrugs-18-00278-s001.zip › Figure S1.pdf]

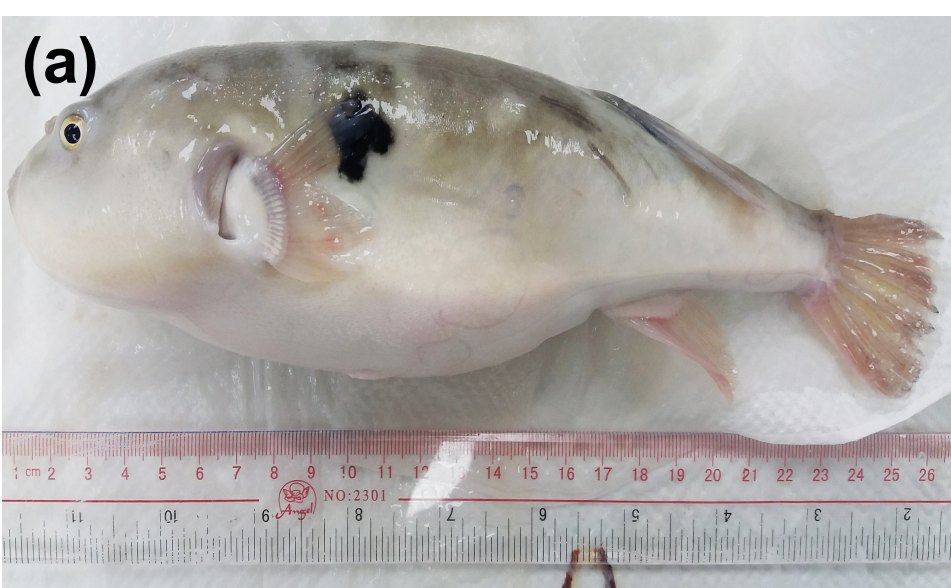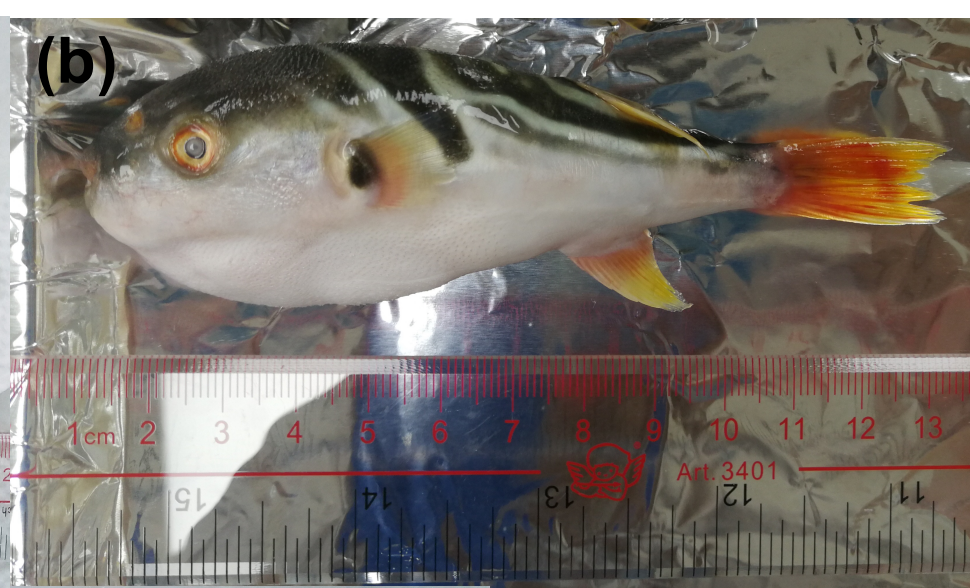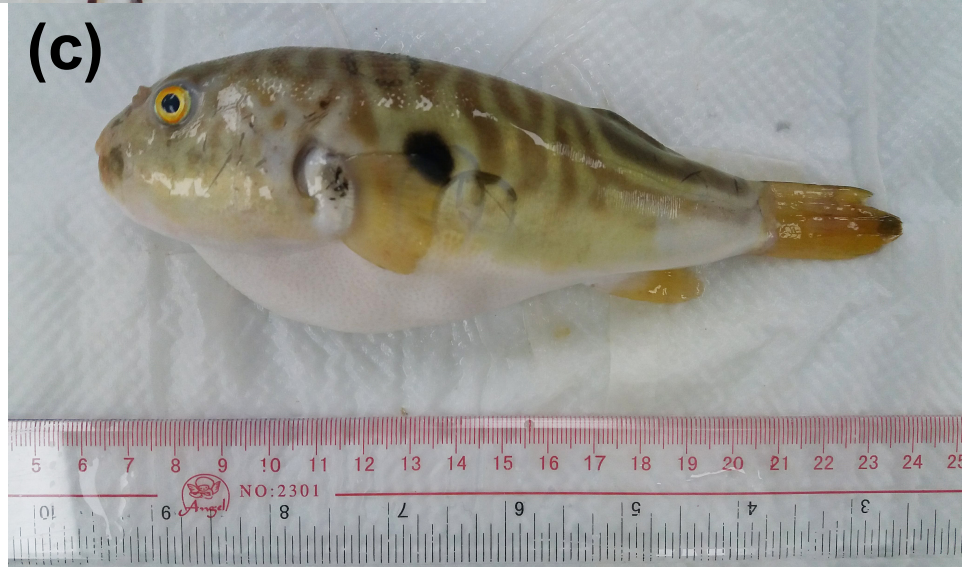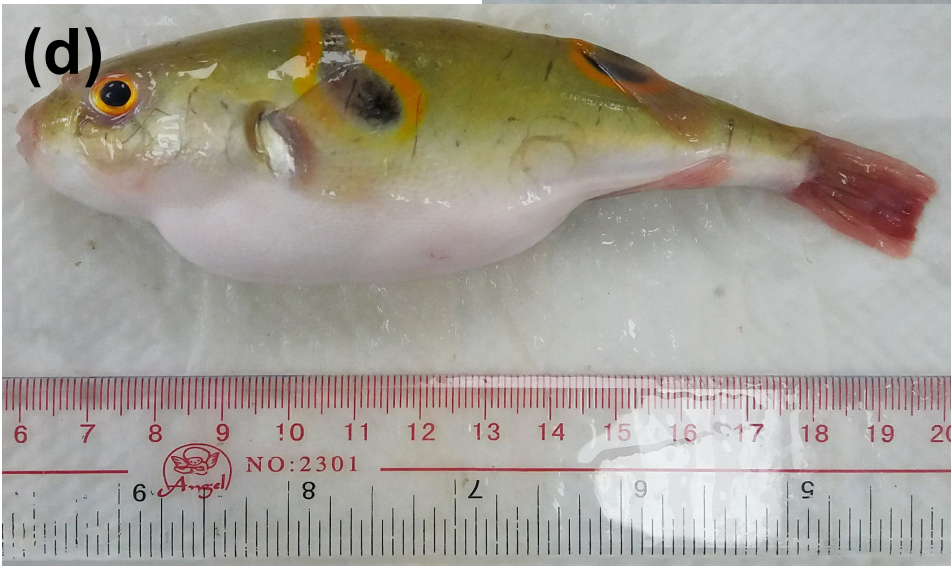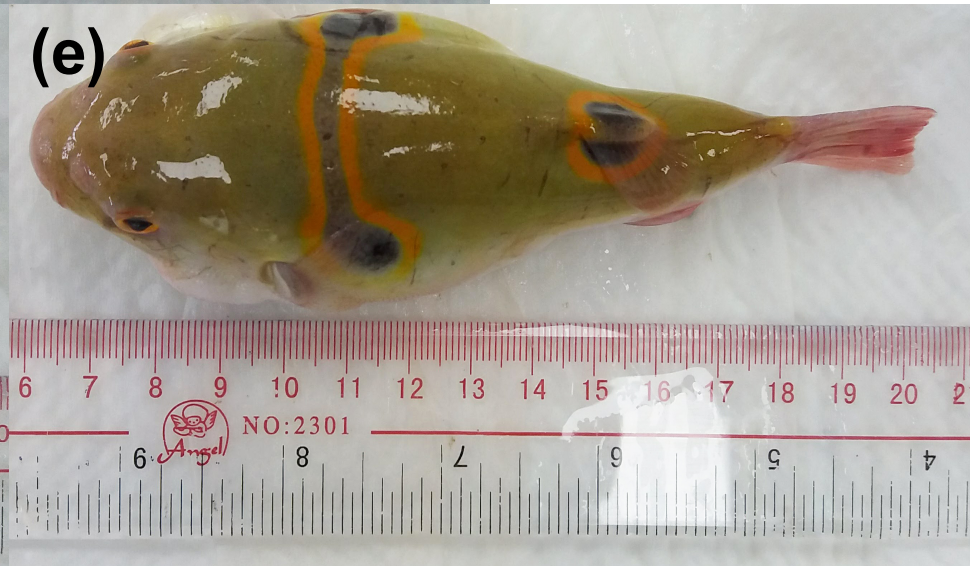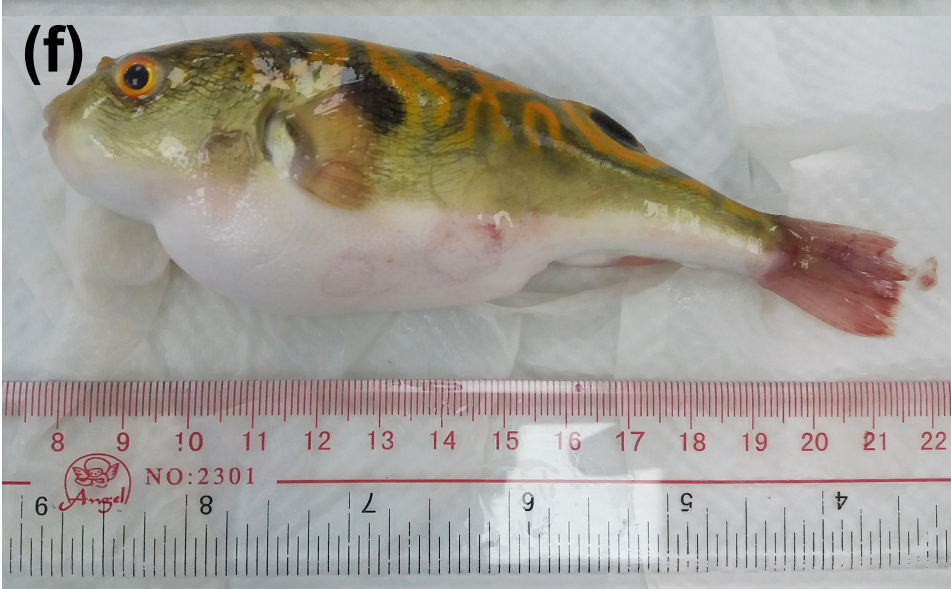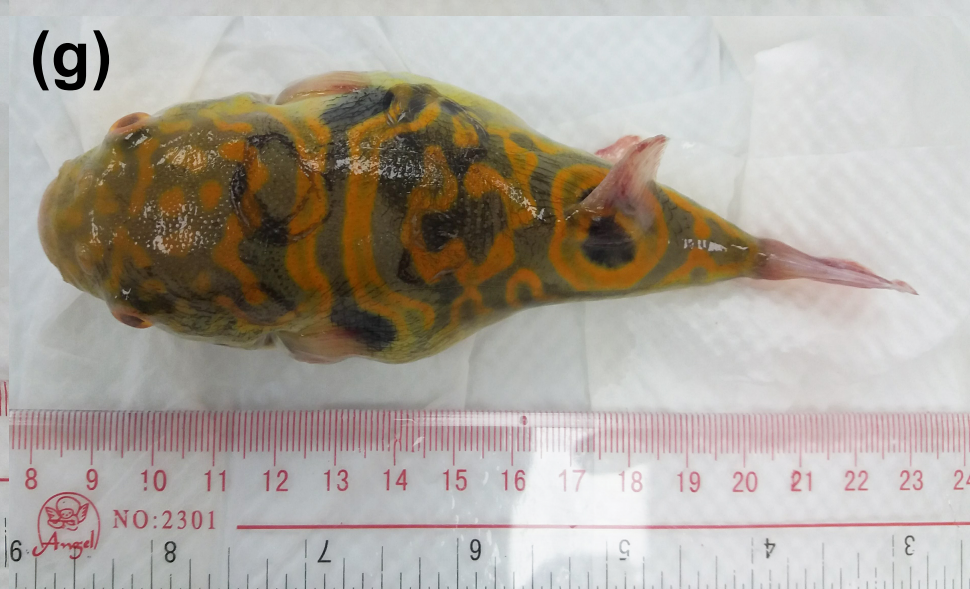

Supplement: Supplementary file 1 [file marinedrugs-18-00278-s001.zip › Figure S2.pdf]
